# Molecular Epidemiology of Multi-Drug Resistant *Acinetobacter baumannii* Isolated in Shandong, China

**DOI:** 10.3389/fmicb.2016.01687

**Published:** 2016-10-21

**Authors:** Meijie Jiang, Lijuan Liu, Yunhua Ma, Zhijun Zhang, Ning Li, Fusen Zhang, Shuping Zhao

**Affiliations:** ^1^The Department of Clinical Laboratory, Tai'an City Central HospitalTai'an, China; ^2^The Department of Hematology, Laiwu City People's HospitalLaiwu, China; ^3^The Department of Clinical Laboratory, Zoucheng City People's HospitalZoucheng, China; ^4^The Department of Preventive Veterinary, College of Veterinary Medicine, Shandong Agricultural UniversityTai'an, China; ^5^Intensive Medicine, Tai'an City Central HospitalTai'an, China

**Keywords:** hospitals, multi-drug resistant *Acinetobacter baumannii*, molecular epidemiology, pulsed-field gel electrophoresis, multilocus sequence typing

## Abstract

*Acinetobacter baumannii* is an emerging nosocomial pathogen prevalent in hospitals worldwide. In order to understand the molecular epidemiology of multi-drug resistant (MDR) *A. baumannii*, we investigated the genotypes of *A. baumannii* isolated from 10 hospitals in Shandong, China, from August 2013 to December 2013, by pulsed field gel electrophoresis (PFGE) and multilocus sequence typing (MLST). Antimicrobial resistance genes were analyzed by PCR and DNA sequencing. By PFGE analysis, we discovered 11 PFGE types in these 10 hospitals. By MLST, we assigned these isolates to 12 sequence types (STs), 10 of which belong to the cloning complex CC92, including the prevalent ST369, ST208, ST195, and ST368. Two new STs, namely ST794 and ST809, were detected only in one hospital. All isolates of the MDR *A. baumannii* were resistant to carbapenem, except 2 isolates, which did not express the *bla*_OXA-23_ carbapenemase gene, indicating *bla*_OXA-23_ is the major player for carbapenem resistance. We also discovered *armA* is likely to be responsible for amikacin resistance, and may play a role in gentamicin and tobramycin resistance. *aac(3)-I* is another gene responsible for gentamicin and tobramycin resistance. In summary, we discovered that the majority of the isolates in Shandong, China, were the STs belonging to the CC92. Besides, two new STs were detected in one hospital. These new STs should be further investigated for prevention of outbreaks caused by *A. baumannii*.

## Introduction

*Acinetobater baumannii* has become one of the most important pathogens of nosocomial infection in China (Wang F. et al., 2013; Hu et al., 2014, 2015). The emergence of multi-drug resistant (MDR) *A. baumannii* strains has brought great difficulties to clinical treatment (Peleg et al., 2008; Doi et al., 2009; Munoz-Price et al., 2013). According to a report from the China Antimicrobial Resistance Surveillance System in 2013, *A. baumannii* is the most prevalent bacteria among all gram negative bacilli and 56.0% of *A. baumannii* are resistant to carbapenem (Wang F. et al., 2013). In 2014, the rate of resistance increased to 62% (Hu et al., 2015).

Epidemiological studies of *A. baumannii* have been constructed in Shandong, China (Ying C. et al., 2015), but a province-wide survey has not been performed. Shandong, as one of the richest regions in China, has an efficient and well-developed transportation. Patients in Shandong often visit cities other than their home city to seek an alternative therapy. This raises the concern that hospital-to-hospital transfer of patients may speed the spreading of MDR *A. baumannii*. In October 2013, after one patient infected with MDR *A. baumannii* was transferred from a secondary hospital to the emergency ward of Hospital-1, Tai'an, Shandong, China, MDR *A. baumannii* with high homology was constantly detected in the sputum specimens of patients hospitalized in the intensive care unit (ICU) (unpublished data).

**Figure 1:**
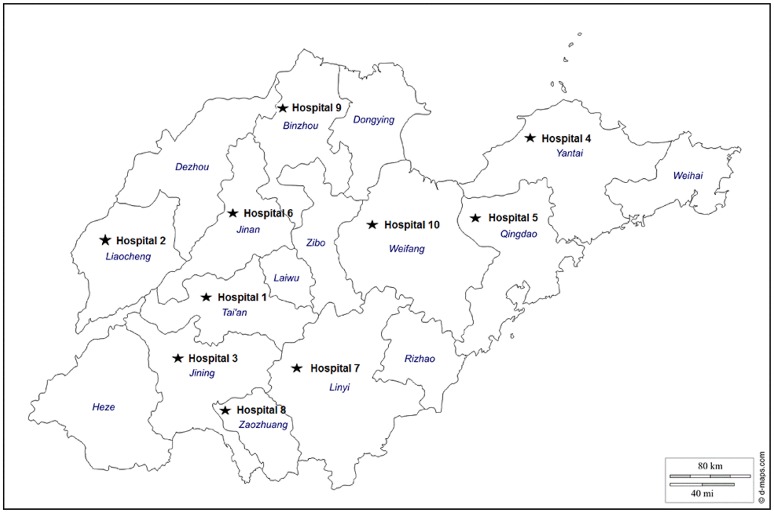
**Geographical locations of the 10 tertiary-care teaching hospitals in Shandong, China**.

The aim of this study was to characterize the epidemiology of the MDR *A. baumannii*, prevalent in Shandong, China. Ten public tertiary-care teaching hospitals (Figure 1) well-spreading in Shandong, China and equipped with advanced medical facilities were selected for this study. The genotypes of the strains of *A. baumannii* isolated from patients were analyzed by pulsed field gel electrophoresis (PFGE) and multilocus sequence typing (MLST) to determine the clonal relatedness, and the genetic characteristics responsible for carbapenem and other antimicrobial resistance mechanisms were identified.

## Materials and methods

### Ethics statement

Samples were collected during a routine checkup by medical professionals. The study was carried out in accordance with the approved guidelines of the Ethics Committee of Tai'an City Central Hospital with written informed consent from all subjects. All subjects gave written informed consent in accordance with the *Declaration of Helsinki*.

### Hospital setting and data collection

This study was performed at 10 public tertiary-care teaching hospitals (termed hospital-1 to -10) located in the major cities with a large population in Shandong Province, China from August 2013 to December 2013 (Figure 1). All of these hospitals equipped with more than 20 beds within the long-stay intensive care unit (ICU) and 1000 beds in other wards, serving more than 10,000 admissions per year. A total of 154 isolates were identified from different clinical specimens of patients administered in either ICU or other wards during this period, including sputum, urine, wound, or cerebrospinal fluid (Table 1).

**Table 1:** **The source of the *A. baumannii* strains and the ward distribution in the 10 tertiary-care teaching hospitals**.

**Hospital**	**No**.	**Sample sources^a^**					**Isolation wards^b^**									
		**SP**	**UR**	**WO**	**AP**	**CF**	**ICU**	**NSW**	**RW**	**NW**	**HSW**	**CW**	**TSW**	**GW**	**BW**	**ED**
1	001–026	26					16	2	1			1		1		5
2	027–041	12		1	1	1	11		1	2				1		
3	042–056	15					14	1								
4	057–071	15					15									
5	072–084	13					13									
6	085–099	11		3		1	6	5		1	2		1			
7	100–109	4		4		2	3	2		1	2				2	
8	110–119	8	2				7	3								
9	120–145	26					14	5	4					1	2	
10	146–154	9					3	3		1				2		
Sum	154	139	2	8	1	4	102	21	6	5	4	1	1	5	4	5

a *SP, sputum; UR, urine; WO, wound; AP, abdominal paracentesis; CF, Cerebrospinal fluid*.

b *ICU, intensive care unit; NSW, Neurosurgery ward; RW, Respiratory ward; NW, Neurology ward; HSW, Hand surgery ward; CW, Cardiology ward; TSW, Thoracic surgery ward; GW, Geriatrics ward; BW, Burn ward; ED, Emergency Department*.

### Bacterial identification and antimicrobial susceptibility test

Bacterial species were identified by using an VITEK-2 bacterial instrument (BioMerieux, Lyons, France) following the manufacturer's instruction. Antimicrobial susceptibility testing was performed by three different methods: the sensitivity of meropenem and cefotaxime was determined by the disk diffusion method; the sensitivity of tigecycline and polymyxin B was determined by the Etest method (AB Biodisk, Solna, Sweden); the sensitivity of other antimicrobial agents was detected using the VITEK-GN13 drug susceptibility card. The criteria of the susceptibility of the GN13 card, meropenem, and cefotaxime were adapted from the Clinical and Laboratory Standards Institute (CLSI; http://clsi.org/standards/). The criteria of the susceptibility of tigecycline were adapted from the United States Food and Drug Administration (http://www.fda.org.uk/sitemap.aspx).

### Pulsed field gel electrophoresis

PFGE was performed as described elsewhere (Ribot et al., 2006). In brief, the chromosomal DNA of *A. baumannii* was digested with 60 U of *ApaI* (Takara, Dalian, China) in a 37°C water bath. With a *Salmonella* serotype Braenderup strain (H9812) digested with *XbaI* (Takara, Dalian, China) as the molecular weight standard, the DNA fragments were separated on a 0.8% agarose gel in 0.5 × TBE using a clamped homogeneous electric field electrophoresis-Mapper XA system (Bio-Rad, California, USA). The experimental conditions were set up as follows: the initial and final switch time of 5 and 20 s, respectively, an included angle of 120° and a gradient of 6 V/cm for 20 h at 14°C. The PFGE images were handled using the Gel Doc software (Bio-Rad, California, USA) according to the operation manual. The PFGE results of 154 strains were disposed using the BioNumerics software (Applied Maths, Belgium) with the uniform marker normalization to record the strip position. A threshold of 85% homology was set to define clonal clustering of PFGE types.

### Multilocus sequence typing

MLST was performed using the method previously described (Bartual et al., 2005). An internal portion of seven housekeeping genes was amplified by PCR, including citrate synthase (*gltA*), DNA gyrase subunit B (*gyrB*), glucose dehydrogenase B (*gdhB*), homologous recombination factor (*recA*), 60-kDa chaperonin (*cpn60*), glucose-6-phosphate isomerase (*gpi*), RNA polymerase σ^70^ factor (*rpoD*), and phospho-glucomutase (*pgm*). PCR experiments were carried out according to a previously described method (Bartual et al., 2005). DNA sequences were analyzed using the MLST database for *A. baumannii* (http://pubmlst.org/abaumannii) and accordingly the sequence types (STs) were assigned.

### Detection of drug resistance genes

DNAs were extracted and amplified by PCR. The primers were designed as previously described (Zhi et al., 2005; Shen et al., 2008; Yang et al., 2011) for the related resistance genes of carbapenemases, including *bla*_IMP_, *bla*_KPC_, *bla*_NDM-1_, *bla*_OXA-23_, *bla*_OXA-24_, *bla*_OXA-48_, *bla*_OXA-50_, *bla*_OXA-51_, *bla*_OXA-55_, *bla*_OXA-58_, *bla*_OXA-60_, and *bla*_OXA64_, for the 16s methylase, including *armA* and *rmtB*, and for the aminoglycoside resistance genes, including *aac(3)-I, aac(3)-II, aac(3)-III, aac(3)-IV, aac(6*′*)-I, aac(6*′*)-II, aph(3*′*)-VI, ant(3*″*)-I*, and *ant(2*′*)-I*. The primers for *bla*_*NDM*−1_ were designed based on the gene sequence announced by the China Disease Prevention and Control Center (Jia et al., 2014).

### Sequence analysis

The positive products of PCR were sequenced by Shanghai Sunny Biotechnology Co., Ltd. (Shanghai, China), and the sequences were compared to the Genbank database.

## Results

From August 20, 2013 to December 20, 2013, we identified 154 strains of *A. baumannii*, isolated from clinical cases in 10 public tertiary-care teaching hospitals in Shandong Province, China (Table 1). *A. baumannii* was initially isolated from sputum (139), urine (2), wound (8), abdominal paracentesis (1), and cerebrospinal fluid (4). Hospital-1 and Hospital-9 were the places where most isolates were identified, with 26 strains in each. More than 66.2% of the isolates (102) were isolated from ICU. In Hospital-4 and -5, ICU was the only place where *A. baumannii* was found.

### PFGE

**Figure 2:**
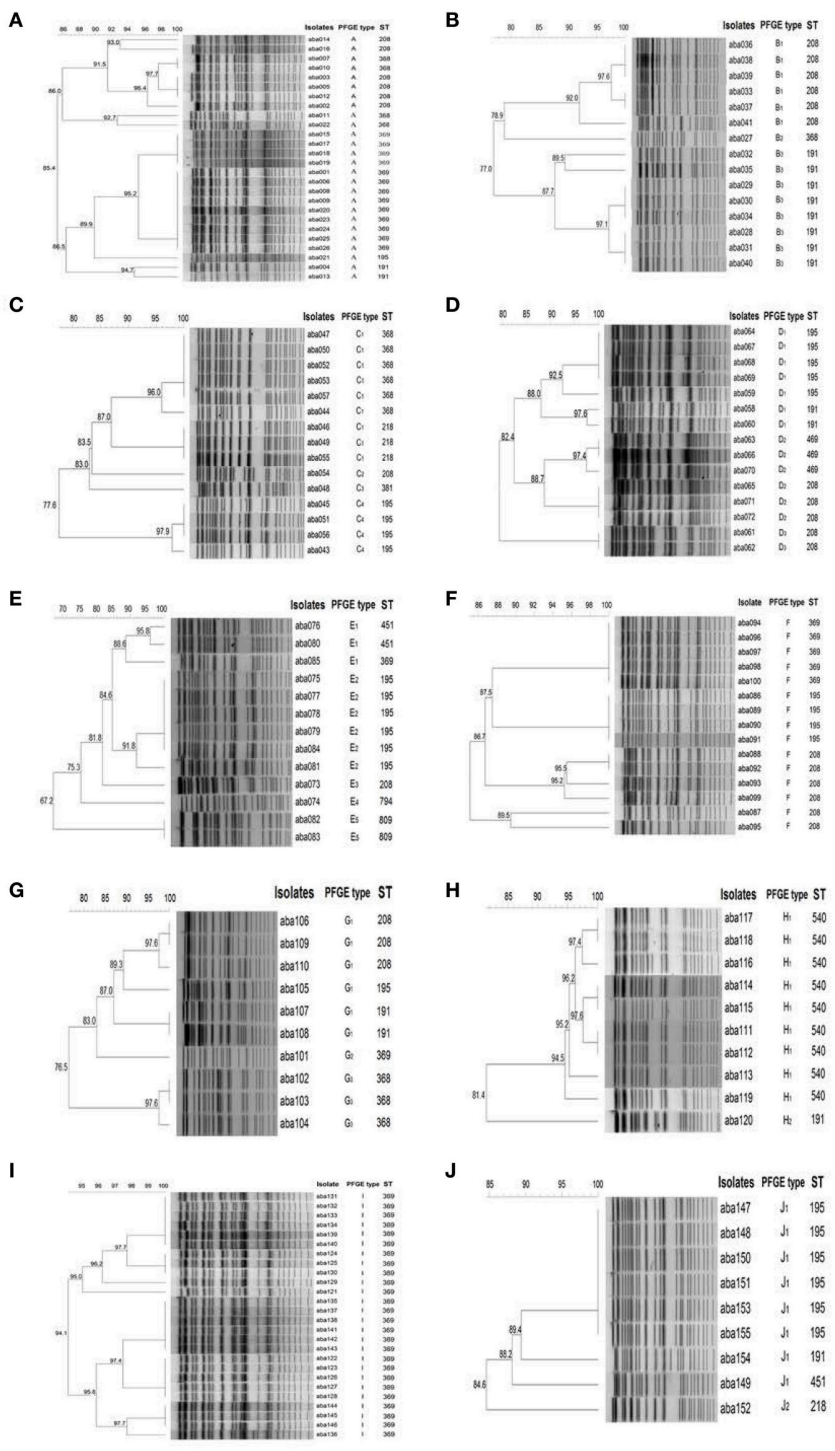
***ApaI* PFGE profiles of representative *A. baumannii* Isolates. (A–J):** isolates from Hospital-1 to -10, respectively. The 85% was set as a cutoff to define PFGE types. The dendrogram was generated by the BioNumerics software.

The *A. baumannii* isolates were assigned to different PFGE types (Figure 2). The isolates in some hospitals, such as Hospital-1, -6, and -9, only had one PFGE type, while in other hospitals, such as Hospital-5, the isolates had as many as five PFGE Types. The PFGE types were further classified to clonal types when the genetic similarity is higher than 85%. The isolates from Hospital-1, -6, and -9 represented one clonal type (Figures 2A,F,I); hospital-8 and -10 had two clonal types (Figures 2H,J); three clonal types were found in hospital-2, -4, and -7 (Figures 2B,D,G); and hospital-3 and -5 had four and five clonal types, respectively (Figures 2C,E).

### MLST

**Figure 3:**
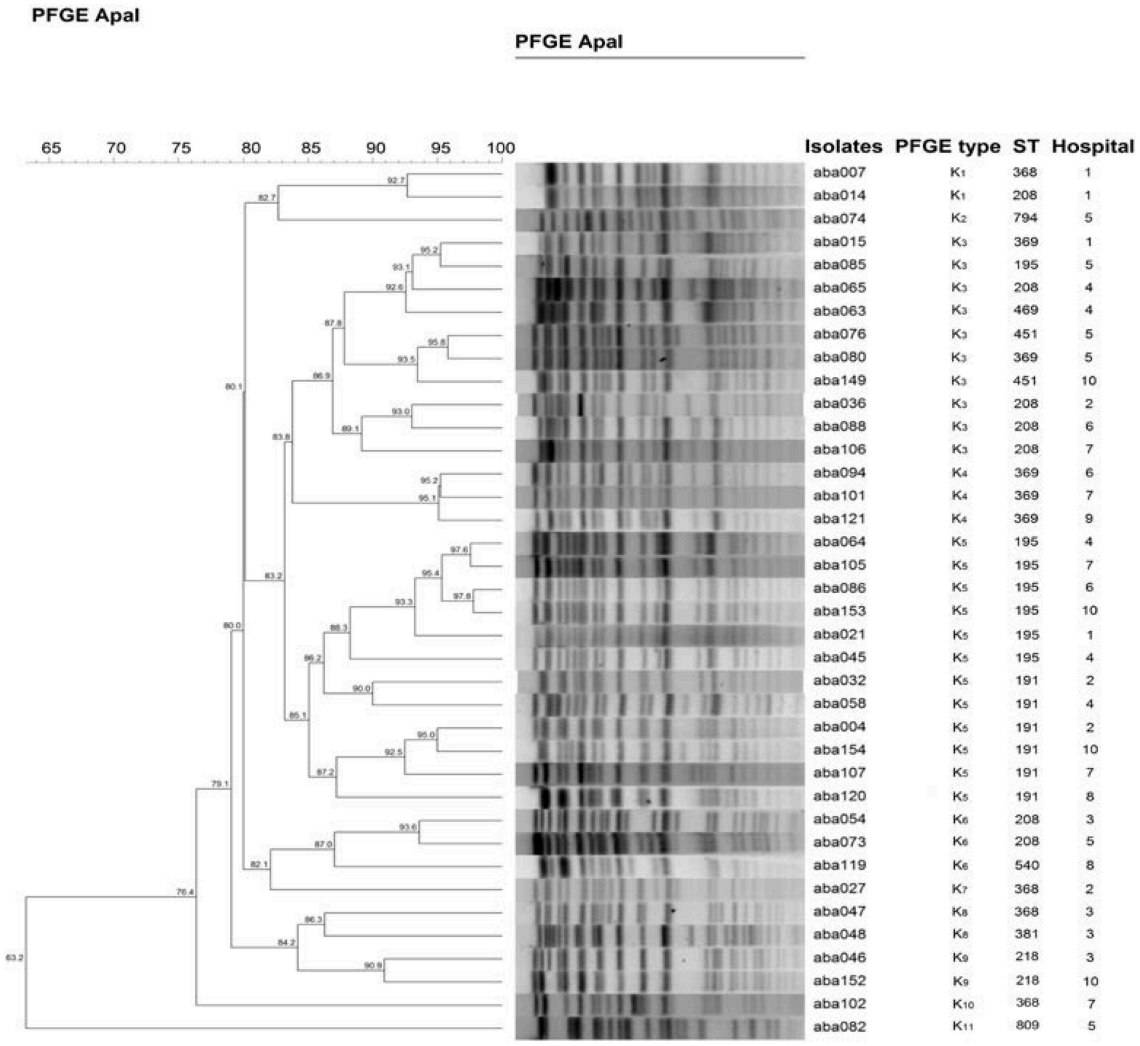
**Dendogram of *A. baumannii* strains selected from 10 hospitals**. At least one representative of all different STs obtained from each hospital was selected to make the dendogram. The 85% was set as a cutoff to define PFGE types. The dendrogram was generated by the BioNumerics software.

A total of 12 STs were designated for the isolates by the MLST analysis, including ST369 (29.9%), ST208 (18.2%), ST195 (17.5%), ST191 (10.4%), ST368 (9.1%), ST540 (5.8%), ST218 (2.6%), ST469 (1.9%), ST451 (1.9%), ST809 (1.3%), ST794 (0.65%), and ST381 (0.65%; Table 2). ST369 is the most representing ST, however, it mainly appeared in two hospitals, Hospital-1 (13) and Hospital-9 (26). ST208 and ST195 were the widest distributed STs, both of which appeared in seven of these 10 hospitals. None of the STs has been found in all hospitals. On the contrary, some STs only appeared in one particular hospital. For example, nine ST368 and one ST381 were found only in Hospital-8 and Hospital-3, respectively. Among these 12 STs, 10 STs (except ST809 and ST794) have been reported belonging to the prevalent cloning complex CC92 (Runnegar et al., 2010). The other two STs, namely ST794 and ST809, were identified as new STs, only appeared in Hospital-5. They are singleton, and do not belong to any clone complex. To make a cross-hospital comparison of the isolates, representatives of different STs obtained from each hospital were selected to plot the dendogram (Figure 3). Using the genetic similarity >85% as a cutoff, 38 isolates can be classified into 11 PFGE types.

**Table 2:** **The distribution of STs of MDRAB in each hospital**.

**Hospital**	**MLST**											
	**ST 369**	**ST 208**	**ST 195**	**ST 191**	**ST 368**	**ST 540**	**ST 218**	**ST 469**	**ST 451**	**ST 809**	**ST 794**	**ST 381**
Hospital-1	13	6	1	2	4	–	–	–	–	–	–	–
Hospital-2	–	6	–	8	1	–	–	–	–	–	–	–
Hospital-3	–	1	4	–	6	–	3	–	–	–	–	1
Hospital-4	–	5	5	2	–	–	–	3	–	–	–	–
Hospital-5	1	1	6	–	–	–	–	–	2	2	1	–
Hospital-6	5	6	4	–	–	–	–	–	–	–	–	–
Hospital-7	1	3	1	2	3	–	–	–	–	–	–	–
Hospital-8	–	–	–	1	–	9	–	–	–	–	–	–
Hospital-9	26	–	–	–	–	–	–	–	–	–	–	–
Hospital-10	–	–	6	1	–	–	1	–	1	–	–	–
Sum	46	28	27	16	14	9	4	3	3	2	1	1

“*–,” no ST was detected in this hospital*.

### Antimicrobial susceptibility testing

The antimicrobial susceptibility profiles of 154 *A. baumannii* strains isolated from August 20, 2013 to December 20, 2013 are shown in Table 3, most of which showed a multiple-antimicrobial-resistant phenotype. All strains were resistant to piperacillin/tazobactam, cefepime, ceftazidime, cefotaxime, ceftriaxone, and piperacillin. A total of 152 out of 154 isolates were resistant to the carbapenems tested, namely imipenem and meropenem. One hundred and thirty eight strains were resistant to levofloxacin and ciprofloxacin. None of the 154 strains were resistant to polymyxin B, and only 10 isolates were resistant to tigecycline. Other relatively effective antibiotics include amikacin, and trimethoprim/sulfamethoxazole, which showed antimicrobial activity against 11 and 12 isolates, respectively.

**Table 3:** **Antimicrobial susceptibility profiles of *A. baumannii* isolates**.

**Antibiotic**	**Resistant**		**Intermediate**		**Susceptible**	
	**Number**	**Rate (%)**	**Number**	**Rate (%)**	**Number**	**Rate (%)**
imipenem	152	98.7	0	0.0	2	1.3
meropenem	152	98.1	0	0.0	2	1.3
piperacillin/ tazobactam	154	100	0	0.0	0	0.0
cefepime	154	100	0	0.0	0	0.0
ceftazidime	154	100	0	0.0	0	0.0
cefotaxime	154	100	0	0.0	0	0.0
ceftriaxone	154	100	0	0.0	0	0.0
levofloxacin	138	89.6	13	8.4	3	1.9
ciprofloxacin	138	89.6	13	8.4	3	1.9
amikacin	143	92.9	0	0.0	11	7.1
tobramycin	145	94.2	0	0.0	9	5.8
gentamicin	145	94.2	0	0.0	9	5.8
piperacillin	154	100	0	0.0	0	0.0
tigecycline	10	6.5	21	13.6	123	79.9
Trimethoprim/ sulfamethoxazole	142	92.2	0	0.0	12	7.8
polymyxin B	0	0.0	0	0.0	154	100

### Carbapenemase genes

The genes of *bla*_IMP_, *bla*_KPC_, *bla*_NDM-1_, *bla*_OXA-24_, *bla*_OXA-48_, *bla*_OXA-50_, *bla*_OXA-55_, *bla*_OXA-58_, and *bla*_OXA-60_ were not detected in this study. As shown in Table 4, the *bla*_OXA-51*-like*_ genes were detected in all 154 MDR *A. baumannii* isolates, and the *bla*_OXA-23_ gene was found in 152 isolates. The isolates had different *bla*_OXA-51*-like*_ genes; 87 strains had the *bla*_OXA-66_ gene and the other 67 strains had the *bla*_OXA-197_ gene (Table 4).

**Table 4:** **Distribution of the carbapenemase genes, aminoglycoside genes, carbapenem resistance, and aminoglycoside antimicrobial resistance of the isolates**.

**Strains No**.	***bla*_OXA-23_**	**Carbapenemase gene**		**Carbapenem resistance**		**Antimicrobial resistant genes of aminoglycoside**					**Aminoglycoside antimicrobial resistance spectrum**		
		***bla*_OXA-51_*a***		**Imipenem**	**Meropenem**	***armA***	***aac(6′)-I***	***ant(3″)-I***	***aac(3)-I***	***aac(3)-IV***	**amikacin**	**gentamicin**	**tobramycin**
		***bla*_OXA-66_**	***bla*_OXA-197_**										
25	+	+	−	R	R	+	−	−	−	−	R	R	R
3	+	+	−	R	R	−	−	+	−	−	S	S	S
39	+	+	−	R	R	+	+	+	−	−	R	R	R
2	+	+	−	R	R	+	−	+	−	−	R	R	R
3	+	+	−	R	R	+	−	+	+	−	R	R	R
9	+	+	−	R	R	+	+	+	+	−	R	R	R
2	+	+	−	R	R	−	−	−	−	−	S	S	S
1	+	+	−	R	R	−	−	+	+	−	S	R	R
1	+	+	−	R	R	+	+	+	−	+	R	R	R
1	−	+	−	S	S	+	−	+	+	−	R	R	R
1	−	+	−	S	S	+	+	+	+	−	R	R	R
4	+	−	+	R	R	−	−	−	−	−	S	S	S
13	+	−	+	R	R	+	+	+	+	−	R	R	R
1	+	−	+	R	R	−	−	+	+	−	S	R	R
4	+	−	+	R	R	+	−	−	−	−	R	R	R
28	+	−	+	R	R	+	+	+	−	−	R	R	R
16	+	−	+	R	R	+	−	+	+	−	R	R	R
1	+	−	+	R	R	+	−	+	−	−	R	R	R
Sum	152	87	67			143	91	119	45	1			

“*+” indicates the gene was detected; “−” indicates the gene was not detected; “R” indicates the strain was resistant to the antibiotic; “S” indicates the strain was susceptible to antibiotic. ^a^bla_OXA-66_ and bla_OXA-197_ are allelic variants of intrinsic bla_OXA-51_*.

### Detection of the 16s methylation enzymes and aminoglycoside resistance genes

As shown in Table 4, 143 strains had the 16s methylation enzyme *armA* and the number of strains encoding other aminoglycoside resistance genes, namely *aac(6*′*)-I, ant(3*″*)-I, aac(3)-I*, and *aac(3)-IV*, were 91,119,45 and 1, respectively. The genes of *rmtB* and *aac(3)-II, aac(3)-III, and ant(2*″*)-I* were not detected. The presence of *armA* is particularly important for amikacin resistance. All 11 strains without *armA* were susceptible to amikacin (Table 4). Out of these 11 strains, two strains containing both *aac(3)-I* and *ant(3*″*)-I* were gentamicin and tobramycin resistant. However, the strain only containing the *ant(3*″*)-I* gene was not resistant to aminoglycoside antimicrobial.

## Discussion

In this study, we investigated the genetic diversity of the *A. baumannii* isolates from 10 tertiary-care teaching hospitals in Shandong, China. All of these hospitals are located in an urban area serving more than 1 million residents and equipped with advanced medical facilities, making them a great fit for the purpose of this study. Our results show that 104 of 154 *A. baumannii* were isolated from patients in ICU, which is consistent with previous reports (Jiang et al., 2013, 2014). It has been reported that mechanical ventilation of ICU is a risk factor for cross infection of *A. baumannii* (Raka et al., 2009; Chaulagain et al., 2012). MDR *A. baumannii* may cause severe respiratory infection symptoms, aggravating the medical conditions of patients in ICU.

The isolates were assigned to 11 PFGE types determined by PFGE (Wang X. et al., 2013). In hospitals like Hospital-6 and Hospital-9, only one PFGE type was found for 15 and 26 isolates, respectively. Using the genetic similarity >85% as a cutoff, we were able to classify the PFGE types into clonal groups to further illustrate the relatedness of these isolates. For example, the isolates in Hospital-1 have a genetic similarity >85% and they were classified into the same clonal group.

MLST is another discriminatory typing method for *A. baumannii*. By MLST, we were able to assign the isolates to 12 STs, which were correlated with the clonal distribution by PFGE (Adams-Haduch et al., 2011). Among the 12 STs, 10 STs belong to the CC92, ST794, and ST809 were identified as new STs, which only appeared in Hospital-5. The main MLST types of *A. baumannii* were ST92 and ST75 in many countries in Asia and the Pacific region, such as Australia, China, India, Japan, and South Korea (Kamolvit et al., 2015). A collection of 398 strains of *A. baumannii* were isolated from seven regions in southern China over the period from January 2012 to June 2012. ST208 was the major type among them, followed by ST191 and ST729 (Ying J. et al., 2015). Overall, the predominant STs of MDR *A. baumannii* might be different in different hospitals, areas, and countries. In our study, we identified ST208 and ST191, which are single-locus variants of ST92. However, we did not observe any ST92, ST75, and ST729.

We discovered ST369 was transmitted by a patient transferred from a secondary hospital to Hospital-1, supporting the notion of the inter-hospital transmission of *A. baumannii* (unpublished data). The common STs prevalent in these hospitals, including ST369, ST208, ST195, ST191, and ST368, may also be a result of inter-hospital transmission (Figure 3). Among these STs, three, namely ST369, ST368, ST195, have been isolated in the Southwest Hospital of Chongqing, China (Huang et al., 2014). Hospital infection control department should not only pay attention to the spreads of drug resistant bacteria in hospital, but also should focus on the spreads of drug resistant strains among hospitals. Among the STs identified, ST794 and ST809 were new STs only identified in Hospital-5. Hospital-5 is located in Qingdao, one of the hottest tourist destinations in China. These isolates may be evolved from local strains, but we cannot rule out the possibility of transmission from tourists. Such transmission has previously demonstrated by the case of *bla*_NDM-1_, which spread from India to Swedish by a tourist (Macfadden et al., 2015). Close attentions should be paid toward these new STs to avoid further transmission between hospitals.

The carbapenemase encoding genes were investigated to decipher the mechanism of carbapenem resistance. Our results show that lacking of the *bla*_OXA-23_ gene led to both imipenem and meropenem susceptible of the isolates, indicating the *bla*_OXA-23_ gene plays an important role in regulating carbapenem resistance. *A. baumannii* carrying the *bla*_OXA-23_ gene distributes widely in many regions in China (Ruan et al., 2013). It has also shown the *bla*_OXA-23_ gene was one of the most common genes in many other countries, such as Saudi Arabia, the United Arab emirates, Oman, Qatar, Bahrain, and Kuwait (Zowawi et al., 2015). The carbapenem resistance of *A. baumannii*, is related to the production of the *OXA-23* type carbapenemases (Runnegar et al., 2010; Mosqueda et al., 2013). In the isolates where the *bla*_OXA-23_ gene was absent, the *bla*_OXA-66_, gene, an allelic variant of intrinsic *bla*_OXA-51_, was expressed; the isolates were carbapenem susceptible. Interestingly, one study has shown that *A. baumannii* isolates only containing the *bla*_OXA-66_ gene were susceptible to carbapenem, but when *bla*_OXA-66_ gene is converted to *bla*_OXA-82_, the *A. baumannii* isolates become carbapenem resistant (Zander et al., 2013). Other *bla*_OXA_ genes, such as *bla*_OXA-40_, can be also involved in the process of carbapenem resistance (Héritier et al., 2003).

Genes responsible for aminoglycoside resistance were also investigated. Multiply genes were involved in the regulation of the resistance pathway for aminoglycosides. One gene, *armA* was particularly important for amikacin resistance. Without the presence of *armA*, the strains were amikacin susceptible. *armA* may play an important role in gentamicin and tobramycin resistance too, as shown in the 29 strains which only expressed the *armA* gene. However, other genes may also be involved in gentamicin and tobramycin resistance, such as *aac(3)-I*. In five strains, where *armA* was not expressed, when only the *ant(3*″*)-I* gene was expressed, the isolates were gentamicin and tobramycin susceptible; when both *ant(3*″*)-I and aac(3)-I* were expressed, the isolates were gentamicin and tobramycin resistant, indicating *aac(3)-I* is responsible for gentamicin and tobramycin resistance in these strains without *armA*. Our results are in consistent with previous studies, which showed the *armA* gene is responsible for the resistance to aminoglycoside antibiotic (Yokoyama et al., 2003; Liu et al., 2012). The roles of the other two genes, namely *aac(6*′*)-I* and *aac(3)-IV* are not clear, since we did not obtain strains with the presence of these two genes alone. Out of the 154 isolates, 10 were resistant to tigecycline. Tigecycline, as well as Polymyxin B, is still one of the most effective therapeutic options in our study, which is consistent with previous studies (Zavascki et al., 2007; Karageorgopoulos et al., 2008; Kassamali et al., 2015). Further investigation should be carried out to decipher the underlying mechanism of the antibiotic resistance of *A. baumannii*.

In summary, we investigated the molecular epidemiology of the MDR *A. baumannii* isolated from 10 public tertiary hospitals in Shandong, China. Our results provide an in-depth analysis of the genetic variability of the *A. baumannii* strains in this region. PFGE and MLST are two commonly used typing methods, by which, we were able to understand not only the evolution of the strains within one given hospital, but also the hospital-to-hospital transmission. Our results show that *bla*_OXA-23_ is the major player for carbapenem resistance. In addition, *armA* is likely to be responsible for amikacin resistance, and may play a role in gentamicin and tobramycin resistance, and *aac(3)-I* is another gene responsible for gentamicin and tobramycin resistance. By the epidemiological study, we hope to pave the road for the establishment of an effective drug resistance monitoring system, and prevention of further spread and outbreaks of the MDR *A. baumannii*.

## Author contributions

MJ performed the main experiment, analyzed data and wrote the manuscript. LL, YM, ZZ, NL, and FZ performed the experiment and analyzed data. SZ reviewed the manuscript and approved it.

### Conflict of interest statement

The authors declare that the research was conducted in the absence of any commercial or financial relationships that could be construed as a potential conflict of interest. The reviewer VG and handling editor declared their shared affiliation and the handling editor states that the process nevertheless met the standards of a fair and objective review.
